# CLINICAL PREDICTORS OF CHOLEDOCHOLITHIASIS IN MILD ACUTE BILIARY PANCREATITIS: DEVELOPMENT OF AN EXPLORATORY RISK STRATIFICATION MODEL

**DOI:** 10.1590/S0004-2803.24612026-032

**Published:** 2026-09-28

**Authors:** Bruno Losi ZACHARIAS, Gustavo Correa MACEDO, Pedro de Souza Lucarelli ANTUNES, Vinícius Alves de ANDRADE, Jacqueline Giannini Arantes PERLINGEIRO, José Cesar ASSEF

**Affiliations:** 1 Irmandade da Santa Casa de Misericórdia de São Paulo (ISCMSP), São Paulo, SP, Brasil.; 2 Faculdade de Ciências Médicas da Santa Casa de São Paulo. São Paulo, SP, Brasil.

**Keywords:** Acute pancreatitis, choledocholithiasis, magnetic resonance cholangiopancreatography, endoscopic retrograde cholangiopancreatography, clinical risk score, Pancreatite aguda, coledocolitíase, colangiopancreatografia por ressonância magnética, colangiopancreatografia retrógrada endoscópica, escore de risco clínico

## Abstract

**Background:**

Acute biliary pancreatitis is one of the most common forms of acute pancreatitis and is frequently associated with transient or persistent common bile duct stones. When not promptly recognized, choledocholithiasis may result in severe complications such as cholangitis and recurrent pancreatitis. Early identification of patients at higher risk for concomitant choledocholithiasis, based on clinical and laboratory findings, is essential to guide timely diagnostic and therapeutic interventions while avoiding unnecessary procedures and costs.

**Objective:**

To evaluate clinical and laboratory predictors associated with choledocholithiasis in patients with mild acute biliary pancreatitis and to develop an exploratory risk stratification model based on routinely available clinical variables.

**Methods:**

A retrospective observational cohort study was conducted through review of medical records and imaging studies of patients admitted to the Central Emergency Department of Santa Casa de São Paulo between January 2017 and August 2024 with mild acute biliary pancreatitis according to the revised Atlanta classification.

**Results:**

A total of 630 medical records were screened, and 145 patients with mild acute biliary pancreatitis met inclusion criteria, with the mean age of 48 years. Among the patients, 65.5% were female. Patients with confirmed choledocholithiasis had significantly higher total bilirubin levels at admission and after 48 hours (*P*=0.001). Alkaline phosphatase levels were also significantly higher in the stone group (*P*=0.011), whereas gamma-glutamyl transferase showed no significant difference (*P*=0.115). Gallstone size did not differ between groups (*P*=0.936). A logistic regression model demonstrated approximately 80% accuracy (AUROC 0.80), based on age ≥65 years, day-2 total bilirubin ≥2.4 mg/dL, and alkaline phosphatase ≥183 U/L. Internal validation using bootstrap resamples demonstrated a AUROC of 0.793. The calibration slope was 1.33, with an intercept of 0.24 and Brier score of 0.126.

**Conclusion:**

A simple clinical prediction model based on older age, persistent bilirubin elevation, and elevated alkaline phosphatase demonstrated acceptable discrimination to associate choledocholithiasis in patients with mild acute biliary pancreatitis and reasonable internal calibration after bootstrap validation, with particular usefulness for identifying patients at low probability of choledocholithiasis.

## HIGHLIGHTS

• Age ≥65 years, persistent bilirubin elevation, and elevated alkaline phosphatase were associated with choledocholithiasis.

• A simple clinical score demonstrated acceptable discrimination for risk stratification of choledocholithiasis.

• The model showed greater utility in identifying patients with a low probability of choledocholithiasis.

## RESUMO

**Contexto:**

A pancreatite aguda biliar é uma das formas mais comuns de pancreatite aguda e está frequentemente associada à presença transitória ou persistente de cálculos no ducto biliar comum. Quando não reconhecida prontamente, a coledocolitíase pode resultar em complicações graves, como colangite e pancreatite recorrente. A identificação precoce de pacientes com maior risco de coledocolitíase concomitante, com base em achados clínicos e laboratoriais, é essencial para orientar intervenções diagnósticas e terapêuticas oportunas, evitando procedimentos desnecessários e custos adicionais.

**Objetivo:**

Avaliar os preditores clínicos e laboratoriais associados à coledocolitíase em pacientes com pancreatite aguda biliar leve e desenvolver um modelo exploratório de estratificação de risco baseado em variáveis clínicas rotineiramente disponíveis.

**Métodos:**

Foi realizado um estudo de coorte observacional retrospectivo por meio da revisão de prontuários e exames de imagem de pacientes admitidos no Pronto-Socorro Central da Santa Casa de São Paulo entre janeiro de 2017 e agosto de 2024 com pancreatite aguda biliar leve, de acordo com a classificação revisada de Atlanta.

**Resultados:**

Um total de 630 prontuários foi analisado, e 145 pacientes com pancreatite aguda biliar leve preencheram os critérios de inclusão, com idade média de 48 anos. Entre os pacientes, 65,5% eram do sexo feminino. Os pacientes com coledocolitíase confirmada apresentaram níveis significativamente mais elevados de bilirrubina total na admissão e após 48 horas (*P*=0,001). Os níveis de fosfatase alcalina também foram significativamente maiores no grupo com cálculos (*P*=0,011), enquanto a gama-glutamil transferase não apresentou diferença significativa (*P*=0,115). O tamanho dos cálculos na vesícula biliar não diferiu entre os grupos (*P*=0,936). Um modelo de regressão logística demonstrou aproximadamente 80% de acurácia (AUROC de 0,80), baseado em idade ≥65 anos, bilirrubina total no segundo dia ≥2,4 mg/dL e fosfatase alcalina ≥183 U/L. A validação interna por meio de reamostragens bootstrap demonstrou uma AUROC de 0,793. A inclinação da calibração foi de 1,33, com intercepto de 0,24 e escore de Brier de 0,126.

**Conclusão:**

Um modelo simples de predição clínica, baseado em idade avançada, elevação persistente da bilirrubina e aumento da fosfatase alcalina, demonstrou discriminação aceitável para associar a coledocolitíase em pacientes com pancreatite aguda biliar leve e calibração interna razoável após validação por bootstrap, com utilidade particular para identificar pacientes com baixa probabilidade de coledocolitíase.

## INTRODUCTION

Acute pancreatitis is a sudden inflammatory condition of the pancreas caused by various etiologies. Gallstone disease is the most common cause, accounting for 40-65% of cases, followed by excessive alcohol consumption, hypertriglyceridemia, hypercalcemia, medication use, infections, trauma, and neoplasms^1^. Gallstones may obstruct the biliary tract, triggering bile reflux, pancreatic enzyme activation, and release of inflammatory mediators affecting the pancreas and adjacent tissues^2^.

Acute biliary pancreatitis (ABP) is frequently associated with transient or persistent common bile duct stones. When not promptly identified, choledocholithiasis may lead to severe complications such as cholangitis and recurrent pancreatitis^3^. According to European (ESGE) and American (ASGE) guidelines, suspicion of choledocholithiasis is based on clinical, laboratory, and ultrasonographic findings. However, ABP itself may cause similar laboratory and imaging alterations, acting as a confounding factor^4^.

Advanced imaging modalities play a central role in diagnostic confirmation. Computed tomography (CT) demonstrates a sensitivity around 22.3% in jaundiced patients and 37.5% in non-jaundiced patients^4^. Magnetic resonance cholangiopancreatography (MRCP) reaches sensitivity up to 97%^5^ ^,^ ^6^, and endoscopic ultrasound (EUS) approximately 96%^7^. Interventional methods such as endoscopic retrograde cholangiopancreatography (ERCP) show sensitivity around 80.2% in jaundiced patients, while intraoperative cholangiography (IOC) reaches approximately 87%^8^ ^,^ ^9^.

Advanced diagnostic modalities improve diagnostic accuracy but are limited by cost, availability, and logistical constraints. The increasing use of imaging studies involving ionizing radiation, particularly CT, has enhanced diagnostic reliability but also raised concerns regarding cumulative radiation exposure, especially when examinations are performed without strict clinical indication^10^. A single abdominal CT scan delivers an effective radiation dose of approximately 8-9 mSv^11^. Although the immediate carcinogenic risk is low, cumulative exposure may increase lifetime cancer risk, particularly with repeated imaging and interventional procedures. This risk appears to rise substantially when cumulative doses exceed 55 mSv^12^. In the evaluation and management of choledocholithiasis, additional exposure may occur, as ERCP and IOC contribute approximately 15-20 mSv and 0.18 mSv, respectively^13^ ^,^ ^14^.

The MRCP, however, is not readily available in many high-volume centers, particularly within the Brazilian Unified Health System (Sistema Único de Saúde - SUS). The performance of this imaging modality entails inherent procedural costs, as well as additional expenses related to patient transportation and referral to higher-complexity facilities. These costs are compounded by those associated with hospitalization and same-admission cholecystectomy - the recommended management strategy for patients with ABP - which, within the SUS in the Southeast region of Brazil, ranges from R$986.00 to R$1,070.00^15^. Fewer than 50% of MRI units in Brazil are available within the public health system. There are approximately 17 MRI units per million inhabitants, and this number can be even lower in low-income regions of the country^16^.

The indication for ERCP, beyond its financial burden, also exposes patients to procedure-related risks, including post-ERCP pancreatitis (4.6-9%), bleeding (up to 1.5%), cholangitis (2.5-6%), and perforation (0.5%)^17^.

Several studies have investigated the correlation between clinical, laboratory, and ultrasonographic parameters and the presence of choledocholithiasis. In 2004, Parreira et al.^18^, within the context of a public healthcare institution, demonstrated a positive correlation between bile duct dilation on ultrasonography and the presence of choledocholithiasis diagnosed preoperatively or intraoperatively. Nevertheless, clinical decision-making could not be reliably based solely on these clinicoradiological findings. Therefore, the combined assessment of clinical and laboratory variables may provide a reliable parameter capable of obviating the need for high-cost imaging studies, procedures involving ionizing radiation, or invasive interventions.

## OBJECTIVE

To evaluate clinical and laboratory predictors associated with choledocholithiasis in patients with mild acute biliary pancreatitis and to develop an exploratory risk stratification model based on routinely available clinical variables.

## METHODS

Data collection and analysis were approved by Ethics Committee (CEP/CONEP - CAAE 91447325.2.0000.5479). This study consists of a retrospective cohort based on the review of medical records and imaging studies of patients treated at the Central Emergency Department of Santa Casa de São Paulo between January 2017 and August 2024. Due to the retrospective design, all eligible patients during the study period were included, and no prior sample size calculation was performed.

The inclusion criteria were adult patients (older than 14 years) who were hospitalized with acute pancreatitis of biliary etiology, which diagnosis was confirmed according to the revised Atlanta classification criteria. Patients were excluded if they had acute pancreatitis of other etiologies, chronic pancreatitis, a history of biliary tract tumors or lesions, age under 14 years, or if they developed moderately severe or severe acute pancreatitis, as defined by severity scores (SOFA, APACHE II, and Marshall). Patients with incomplete medical records were also excluded.

The following variables were analyzed: age, sex, total bilirubin levels at admission and during hospitalization follow-up, cholestatic enzymes (gamma-glutamyl transferase and alkaline phosphatase), presence of clinical jaundice, and gallstone size. Choledocholithiasis was considered present when identified on ERCP, MRCP, or IOC.

The correlation between total bilirubin levels at admission and after two days of treatment and the presence of common bile duct stones was assessed in patients with mild ABP and no evidence of severity score deterioration. This analysis was stratified into two groups: patients younger than 65 years and the overall cohort including all ages. A multivariate analysis was also performed to evaluate the association between canalicular enzyme levels, age, sex, and gallbladder stone size and the presence of choledocholithiasis associated with acute biliary pancreatitis.

Statistical analysis was conducted using multivariate parametric methods. For continuous variables, Student’s *t*-test was applied when normal distribution could be assumed (Kolmogorov-Smirnov test, *P*>0.05), and the Mann-Whitney test was used when normality assumptions were not met. Categorical variables were analyzed using the chi-square test or Fisher’s exact test, as appropriate. A significance level of 5% was adopted for rejection (or non-rejection) of the null hypothesis.

Internal validation was performed using bootstrap resampling with 1000 iterations. Model discrimination was assessed by calculating optimism-corrected AUROC values derived from bootstrap samples. Model calibration was evaluated using calibration plots, calibration slope and intercept, and Brier score analysis.

## RESULTS

A total of 630 medical records were reviewed, of which 485 were excluded according to the following criteria (Figure 1): acute pancreatitis of non-biliary etiology; moderately severe or severe disease; absence of active investigation for choledocholithiasis (i.e., no ERCP, MRCP, or IOC as a diagnostic modality); and incomplete medical records.

**FIGURE 1:**
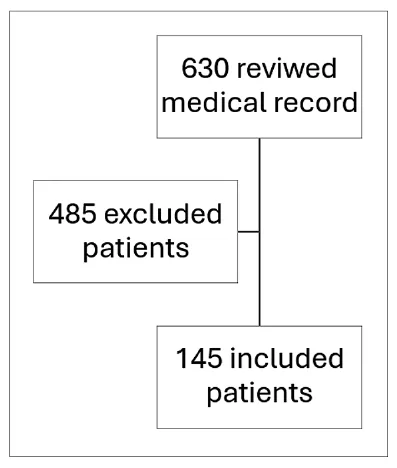
Patients inclusions diagram.

Among the 145 included patients, 65.5% were female, with a mean age of 48 years. The prevalence of confirmed choledocholithiasis was 19.3% (28/145). There was no significant difference in sex distribution between groups (*P*=0.879). A higher prevalence of elderly patients was observed in the choledocholithiasis group (32.1% vs 12.0%; *P*=0.018). The mean age was slightly higher in patients with common bile duct stones (52.9±17 vs 47.5±14 years); however, this difference did not reach statistical significance in continuous variable analysis (*P*=0.083).

The analyzed variables and their correlation with the presence of choledocholithiasis are summarized in Figure 2.

**FIGURE 2:**
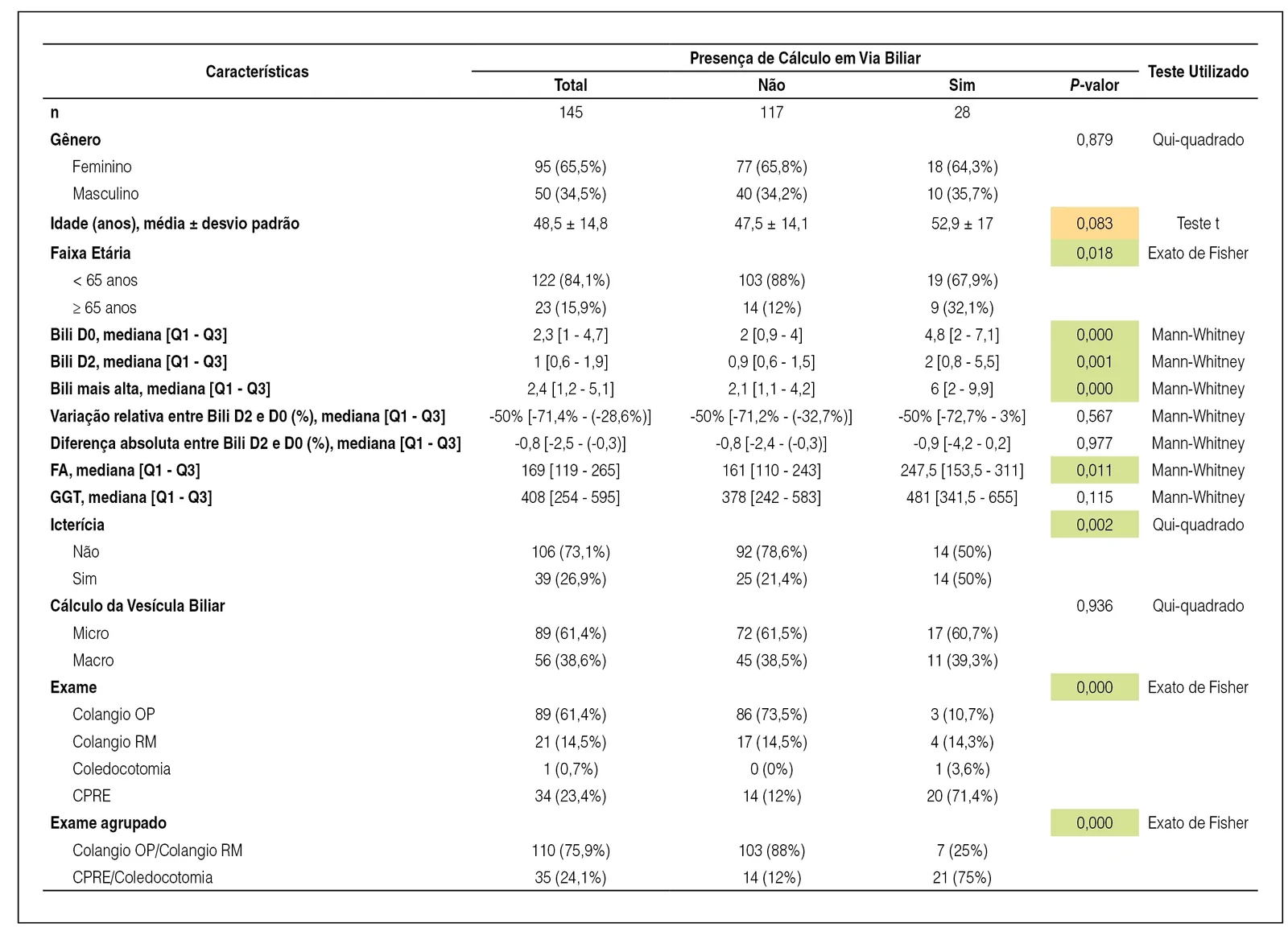
Clinical, laboratory, and radiological data of the analyzed patients and their correlation with the presence of common bile duct stones.

Initial total bilirubin levels (reference range: 0.2-1.3 mg/dL) at admission (D0) were significantly higher in patients with confirmed choledocholithiasis. The median D0 bilirubin in the stone group was 4.8 mg/dL [2.0-7.1], compared to 2.0 mg/dL [0.9-4.0] in the group without stones (*P*<0.001; Mann-Whitney test). After 48 hours of conservative management (D2), bilirubin levels decreased in both groups; however, D2 bilirubin remained significantly higher in the stone group (median 2.0 mg/dL [0.8-5.5] vs 0.9 mg/dL [0.6-1.5] in the non-stone group; *P*=0.001).

The percentage reduction in bilirubin from D0 to D2 was similar between groups (median approximately 50% in both; *P*=0.567). Likewise, the absolute change in bilirubin (D2 - D0) did not differ significantly between groups (median ≈ -0.8 mg/dL in both; *P*=0.977).

Regarding cholestatic enzymes, alkaline phosphatase (reference range: 38-126 U/L) was significantly higher in the stone group, with a median of 247 U/L [154-311] vs 161 U/L [110-243] in the non-stone group (*P*=0.011). Gamma-glutamyl transferase (reference range: 12-52 U/L) levels were also higher in patients with stones (median 481 vs 378 U/L), although with considerable variability and without statistical significance (*P*=0.115).

Clinically, jaundice was present in 50% of patients with choledocholithiasis, compared to 21.4% of those without stones (*P*=0.002). Concerning gallbladder calculi, stone size (microstones <5 mm vs macrostones ≥5 mm) did not differ between groups, with similar proportions of small (~61%) and large (~39%) stones observed in both groups (*P*=0.936).

A logistic regression model was developed to construct a clinical-laboratory score aimed at ruling out choledocholithiasis. The variables included were age (categorical, ≥65 years), total bilirubin at 48 hours (D2 ≥2.4 mg/dL), and alkaline phosphatase (≥183 U/L).

This regression model demonstrated an overall accuracy of approximately 80% for identifying the presence of choledocholithiasis (AUROC = 0.80) (Figure 3) and a specificity of 92% for excluding the diagnosis (Table 1). Internal validation using 1000 bootstrap resamples demonstrated a AUROC of 0.793 (Table 2). The calibration slope was 1.33, with an intercept of 0.24. The Brier score was 0.126 (Figure 4).

**FIGURE 3:**
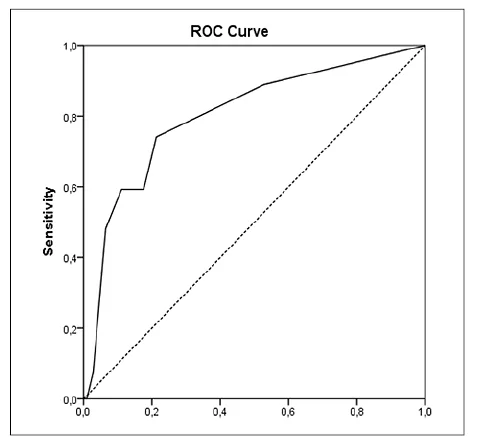
Score AUROC.

**TABLE 1:** Table showing the distribution of patients across score ranges and the corresponding diagnostic performance (PPV, NPV, and p-values).

Score Range	Total (%)	No Stone (%)	Stone (%)	PPV	NPV	***P*-value**
Low Risk (< 300)	100 (69%)	92 (79%)	8 (29%)	0.08	0.92	0.000
Intermediate Risk (300-420)	25 (17%)	18 (15%)	7 (25%)	0.28	0.72	0.266
High Risk (> 420)	20 (14%)	7 (6%)	13 (46%)	0.65	0.35	0.000

**TABLE 2:** Internal validation using bootstrap.

Metric	Value
Apparent AUROC	0.806
Mean bootstrap AUROC	0.805
Mean test AUROC on original cohort	0.792
Estimated optimism	0.013
Optimism-corrected AUROC	0.793

**FIGURE 4:**
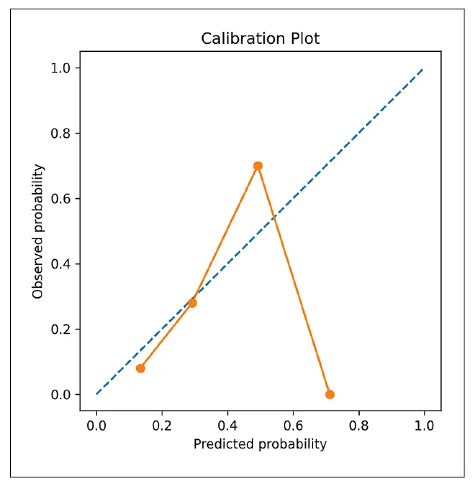
Calibration.

**Final Score Formula:** 210 + 120 × (“Age ≥65 indicator”) + 130 × (“D2 total bilirubin ≥2.4 mg/dL indicator”) + 80 (“Alkaline phosphatase ≥183 U/L indicator”).

## DISCUSSION

Secondary choledocholithiasis is a condition that may be associated with ABP and should be promptly diagnosed and treated to prevent clinical complications, prolonged hospitalization, and consequently increased overall treatment costs for the institution.

Patients with persistent common bile duct stones present with higher initial total bilirubin levels and maintain higher absolute values at 48 hours, although a downward trend is observed regardless of stone persistence.

An initial logistic regression model was constructed including the following variables: age (categorical ≥65 years), total bilirubin at admission (D0 ≥4.3 mg/dL), total bilirubin at 48 hours (D2 ≥2.4 mg/dL), alkaline phosphatase (≥183 U/L), and gamma-glutamyl transferase (≥381 U/L). Although gamma-glutamyl transferase did not demonstrate statistical significance in the univariate comparative analysis (*P*=0.115), it was retained in the initial multivariate model due to potential clinical relevance. After sequential adjustments, gamma-glutamyl transferase was excluded because of lack of significance (*P*>0.9), as was D0 bilirubin, which, despite correlating with the outcome in isolation, did not maintain significance in the combined model (*P*=0.317).

The final model identified three independent predictors of choledocholithiasis: age ≥65 years, D2 total bilirubin ≥2.4 mg/dL, and alkaline phosphatase ≥183 U/L. Each of these variables showed a statistically significant association with the outcome, with elevated odds ratios (Table 1). In summary, elderly patients with persistently elevated alkaline phosphatase and total bilirubin levels after 48 hours exhibited a substantially higher probability of harboring persistent common bile duct stones compared to other patients.

Based on these three final predictors (age, D2 bilirubin, and alkaline phosphatase), a risk score was developed with a potential role as a rule-out tool in clinical practice. The low-risk category (score <300) identified approximately 90% of patients without stones (negative predictive value ~92%), demonstrating a strong discriminatory capacity for excluding choledocholithiasis. The high-risk category (score >420) demonstrated an approximately 65% probability of choledocholithiasis, aligning with guideline definitions of “high risk” (incidence >50%). 

The present study has limitations, including potential biases inherent to retrospective data collection and variability in the quality and completeness of medical records and imaging studies. All patients included in the study underwent active investigation for choledocholithiasis using highly specific diagnostic modalities, namely ERCP, IOC, or MRCP. No discordant findings among these modalities were observed in the study population, suggesting a low risk of verification bias.

Furthermore, patients with ABP and a low probability of associated choledocholithiasis who were hospitalized between 2020 and 2021, during the COVID-19 pandemic, were excluded from the study because they did not undergo any of these three confirmatory examinations, due to institutional efforts to reduce hospital length of stay during this period. This may have created a selection bias, potentially increasing the prevalence of choledocholithiasis in the sample and overestimating the discriminatory performance of the proposed model, including AUROC and predictive values. All other patients hospitalized during the remaining study periods underwent active investigation for choledocholithiasis using at least one of the three diagnostic modalities.

Although the number of outcome events was relatively limited, internal bootstrap validation suggested that severe overfitting was unlikely. Nevertheless, calibration instability was observed in the highest predicted-risk stratum, where the model tended to overestimate the actual probability of choledocholithiasis. This finding is likely related to the limited number of outcome events and the small number of patients within extreme-risk categories, reducing the precision of probability estimates. The relatively high negative predictive value observed among low-risk patients suggests that the model may be more useful as a rule-out tool for identifying patients at low probability of choledocholithiasis rather than as a definitive diagnostic instrument for confirming disease, particularly because positive predictive performance and calibration in higher-risk strata remained less stable.

Therefore, the model should still be interpreted as an exploratory derivation tool requiring prospective external validation before broader clinical implementation.

## CONCLUSION

In patients with mild acute biliary pancreatitis, older age, persistent elevation of bilirubin, and elevated alkaline phosphatase were independently associated with the presence of choledocholithiasis. Based on these variables, a simple clinical prediction model demonstrated acceptable discriminatory capacity that showed usefulness in identifying patients at low probability of choledocholithiasis, suggesting a potential role as a rule-out tool in clinical practice. 

Nevertheless, given the retrospective single-center design and the need for external validation, the proposed score should be considered exploratory and hypothesis-generating until further prospective validation is performed. The score may be particularly relevant in low-complexity settings where access to advanced biliary imaging is limited.

## Data Availability

Data-available-upon-request
